# Use of blue fluorescent protein Electra2 for live-cell imaging in *Dictyostelium discoideum*

**DOI:** 10.17912/micropub.biology.001774

**Published:** 2025-08-22

**Authors:** Hidenori Hashimura, Hibiki Nakagawa, Satoshi Sawai

**Affiliations:** 1 Department of Basic Science, Graduate School of Arts and Sciences, The University of Tokyo, Tokyo, Japan; 2 Research Center for Complex Systems Biology, Universal Biology Institute, The University of Tokyo, Tokyo, Japan

## Abstract

Because of its good spectral separation from green (GFP) and red (RFP) fluorescent proteins, blue fluorescent protein (BFP) is essential for multicolor live cell imaging. However, the commonly used bright mTagBFP2 strongly perturbs the cellular localization of Lifeact, an F-actin marker. As an alternative, we tested the expression of Electra2 in *Dictyostelium* . Both standalone and as a fusion tag to Lifeact, HistoneH1, or the Akt/PKB PH domain, Electra2 showed brightness comparable to that of mTagBFP2, with intracellular localization patterns consistent with those of GFP and RFP. Electra2 is a promising BFP of choice for studying actin and other targets.

**Figure 1. Fluorescence imaging with blue fluorescent protein Electra2 in D. discoideum:**
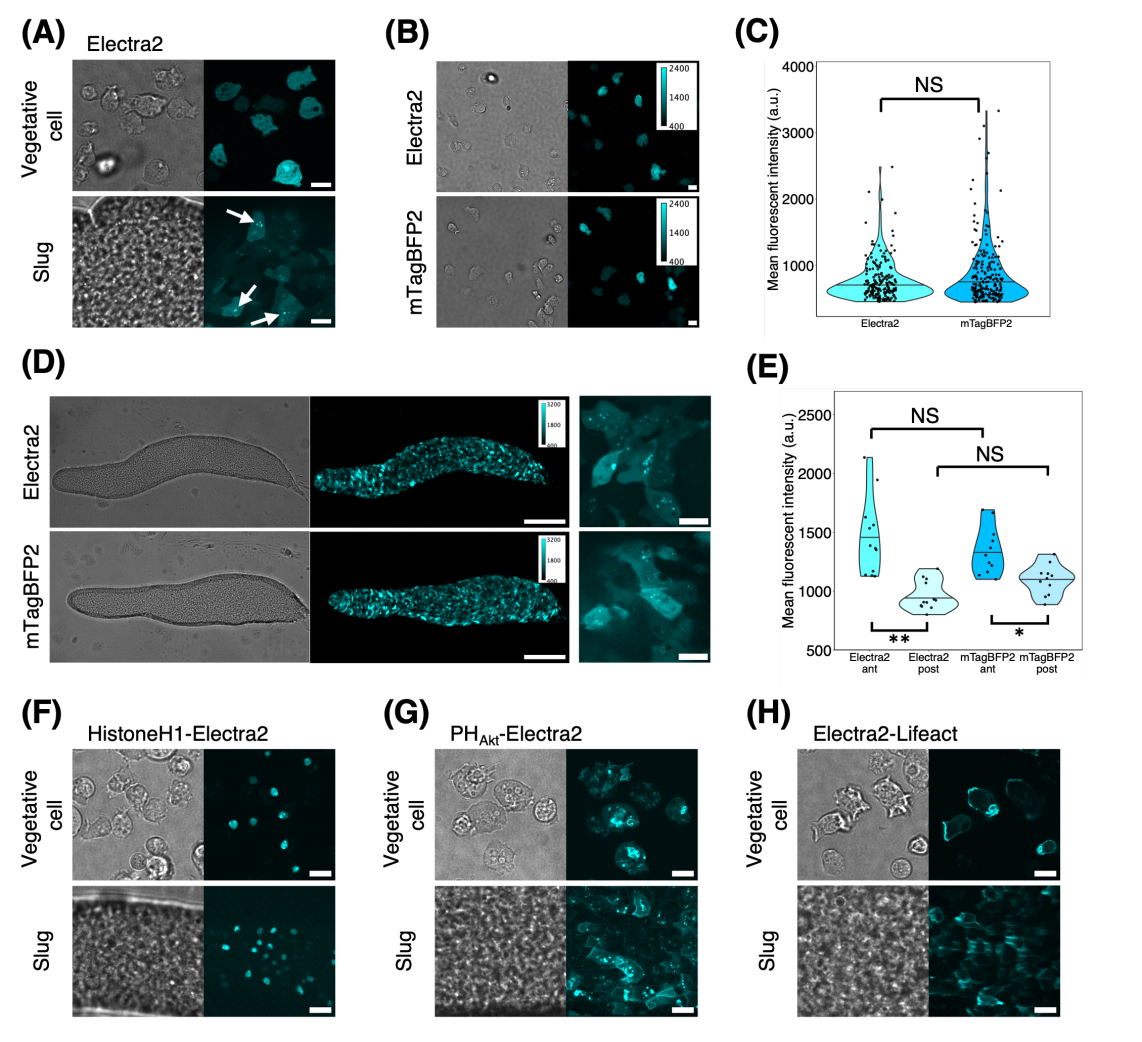
(A) Cells expressing Electra2 under the *act15* promoter. Vegetative cells (upper panel) and slug-stage cells (lower panel). Scale bar, 10 µm. Brightfield (left panels) and fluorescence images (right panels). White arrows indicate Electra2 aggregates. (B) Vegetative cells expressing Electra2 (upper panels) and mTagBFP2 (lower panels) under the *act15* promoter (left: brightfield channel, right: fluorescence channel). Scale bar, 10 µm. (C) Violin plot of the single-cell mean fluorescence intensity distribution of Electra2- and mTagBFP2-expressing vegetative cells (Electra2, n = 190 cells. mTagBFP2, n = 217 cells). The black line is the median. NS: *P* >= 0.05. (D) A slug expressing Electra2 (upper panels) and mTagBFP2 (lower panel) under the *act15* promoter (left: brightfield channel, right: fluorescence channel); magnification low (left panel) and high (right panel). Scale bar, 100 µm (left) and 10 µm (right). (E) A violin plot of the mean fluorescent intensities in the anterior and the posterior region of the slugs (Electra2, n = 12 slugs. mTagBFP2, n = 11 slugs). The black line indicates the median value.**: *P* < 10 ^−4^ . *: *P* < 0.05. NS: not significant ( *P* >= 0.05). (F–H) The brightfield (left panels) and fluorescence images (right panels) of Electra2-expressing vegetative (upper panels) and slug-stage cells (lower panels). The anterior-posterior axis of the slug is from left to right. Scale bar, 10 µm. (F) *act15p* :HistoneH1-Electra2. (G) *coaAp* :PH _Akt_ -Electra2. (H) *act15p* :Electra2-Lifeact.

## Description

With excitation and emission wavelengths that peak around 405 nm and 456 nm, respectively, blue-color fluorescent proteins (BFPs) are becoming essential for multicolor imaging because of their good spectral separation from GFP and RFP as well as compatibility with the light source and filter sets widely used for nuclear DAPI staining. mTagBFP2 derived from eqFP578 in *Entacmaea quadricolor* , one of the brightest BFPs available (Merzlyak et al., 2007; Subach et al., 2011), has been utilized for multi-color fluorescence imaging in various organisms: from budding yeast, *C. elegans* (Sakai et al., 2021; Yemini et al., 2021) to cellular slime mold *Dictyostelium* (Hashimura et al., 2024). A shortcoming of mTagBFP2, despite its high stability and brightness, is that it sometimes shows disparity in intracellular localization when fused to other proteins, most notably F-actin-binding Lifeact (Hashimura et al., 2024; Lee et al., 2013; Vještica et al., 2019) possibly by affecting Lifeact affinity to F-actin. This limitation greatly hinders the choice of FPs for multicolor imaging-based studies involving observations of F-actin and its regulators.

A possible alternative to mTagBFP2 is the bright blue fluorescent protein Electra2 (Papadaki et al., 2022) recently developed from another *E. quadricolor* fluorescent protein eqFP611 (Wiedenmann et al., 2002). Its brightness is comparable to that of mTagBFP2 when expressed in mammalian cells, and it has been successfully applied to label neurons in *C. elegans* , zebrafish, and mouse (Papadaki et al., 2022). To test the performance of Electra2 as a fluorescent probe in *D. discoideum* , we examined its fluorescence when it was expressed alone. During the growth phase of *D. discoideum* cells, Electra2 fluorescence appeared uniformly in the cytosol and nucleus ( Fig. 1A ). Aggregates were visible in slug-stage cells ( Fig. 1A ; arrows). The brightness of Electra2 was indistinguishable from that of mTagBFP2 in both vegetative and slug-stage cells ( Fig. 1B –E). The difference between the anterior and posterior parts of slugs in the fluorescence intensity of mTagBFP2 or Electra2 reflects higher activity of the *act15* promoter in prestalk cells ( Fig. 1E ). These features — bright uniform fluorescence in the vegetative cells and the appearance of aggregates in the developing cells — have also been described for mTagBFP2 (Hashimura et al., 2024).

Next, we expressed Electra2 as a fusion tag. As expected, the fluorescence of Electra2 fused to HistoneH1 (HistoneH1-Electra2) appeared in the nucleus in both vegetative and slug-stage cells ( Fig. 1F ). Electra2 fused to the PH domain of Akt/PKB (Meili et al., 1999) was localized to the expected regions: pinocytic cups in vegetative cells and the front cell-cell contact site in slug-stage cells ( Fig. 1G ). These patterns were similar to those observed for HistoneH1-mTurquoise2 and PH _Akt_ -mTurquoise2 (Hashimura et al., 2024). Electra2-Lifeact appeared throughout the cell cortex and was markedly concentrated in pinocytic cups and the leading edges ( Fig. 1H, upper panel). In slug-stage cells, Electra2-Lifeact was localized to the cell cortex and leading edges ( Fig. 1H, lower panel). These patterns of Lifeact localization were in close agreement with those observed for GFP-, mTurquoise2-, LSSmGFP, and mRFP-Lifeact (Brzeska et al., 2014; Hashimura et al., 2024; Lemieux et al., 2014) as well as for phalloidin staining, (Lemieux et al., 2014) thus circumventing the issue of altered mTagBFP2-Lifeact localization to the cell rear (Hashimura et al., 2024).

Our results demonstrate that in *D. discoideum* cells, Electra2 and its fusion proteins are well expressed and exhibit show localization patterns consistent with those of proteins tagged by other major FPs. These features make Electra2 a desirable BFP for studying the co-localization of F-actin and its regulators, where one is frequently required to swap between compatible FPs with different spectral characteristics. It should be noted that this does not preclude the use of mTagBFP2 for other purposes; for example, it may be advantageous to monitor gene expression owing to its rapid maturation (Subach et al., 2011). For both mTagBFP2 and Electra2, aggregates formation is known to occur in *C. elegans* , zebrafish, and mice, (Papadaki et al., 2022) and as shown here, in developing *D. discoideum* cells. Similar puncta were also reported for mRuby2 (Costantini et al., 2015) , which also derived from eqFP611 (Lam et al., 2012). Although the identity of the aggregates remains to be studied, they may be similar to those of DsRed and its variants in mammalian cells, where they accumulate in the lysosome owing to their resistance to acidity and proteases (Katayama et al., 2008). It is desirable that future BFPs address this aspect to make it truly interchangeable with major green and red FPs.

## Methods

**Plasmids and transformation**

The plasmids and primers used in this study are summarized in the reagents section. For constitutive expression, all vectors used the *act15* promoter with the exception of pDM304_ *coaAp* :PH _Akt_ -Electra2, which used the *coaA* promoter. To obtain N- and C-terminal FP tag vectors, pDM304_ *act15p* :MCS-(GGS) _2_ -Electra2 and pDM304_ *act15p* :Electra2-(GGS) _2_ -MCS, Dicty-codon-optimized DNA for Electra2 (Papadaki et al., 2022) was synthesized and inserted into the BglII or SpeI sites of pDM304 (Veltman et al., 2009) by fusing PCR-generated inserts and linearized vectors with In-Fusion enzyme (In-Fusion Snap Assembly cloning kit, Takara).

The DNA sequence of the codon-optimized Electra2 is as follows: ATGGTAAGTAAAGGAGAAGAATTAATCGAAGAAAATATGCGTATGAAAGTGGTTATGGAAGGATCTGTAAATGGACATCAATTTAAATGTACCGGCGAAGGTGAAGGAAGACCATATGAAGGAGTACAAACAATGAGAATTAAAGTAATTGAAGGTGGTCCATTACCATTTGCTTTTGATATTTTAGCCACTTCATTCTTATTCGGTAGTAAAACTTTTATTAAATACCCTGCAGATATACCAGATTTCTTTGAACAAAGTTTTCCAGAAGGTTTTACCTGGGAAAGAGTTACAAGATATGAAGATGGTGGTGTAGTTACTGTTACTCAAGATACATCACTTGAAGATGGTGGTTTAGTTTATAATGTTAAAGTCCGTGGTGTTAATTTCCATTCAAAAGGTCCAGTAATGCAAAAGAAAACTGAAGGTTGGGAACCATTTACTGAAATGATGTATCCAGCTGATGGTGGTTTAAGAGGTTATACAGATATAGCTCTTAAAGTTGATGGTGGTGGTCATTTACATGCAAATATTGTAACAACATATCGTTCAAAGAAAACAGTCGGTAATATTAAAATGCCAGGTGTTCATGCAGTTGATTATAGATTAGAAAGAATTGAGGAGTCAGATAATGAGACATATGTTGTTTTGAGAGAAGTTGCAGTTGCAAAATATTCAAATTTGGGTGGTGGTATGGATGAATTATTTAAA.

To generate expression vectors for HistoneH1-Electra2 and PH _Akt_ -Electra2, a fragment containing coding sequences of HistoneH1 or the PH domain of Akt/PKB (amino acids 1–111) (Hashimura et al., 2024; Meili et al., 1999) was inserted into the BglII and SpeI cloning sites of pDM304_ *act15p* :MCS-(GGS) _2_ -Electra2. The *coaA* promoter (Hashimura et al., 2024; Paschke et al., 2018) was inserted between the XhoI and BglII sites of pDM304_ *act15p* :PH _Akt_ -Electra2 to generate pDM304_ *coaAp* :PH _Akt_ -Electra2. To obtain an expression vector for Electra2-Lifeact, annealed Lifeact DNA oligos (Riedl et al., 2008) were inserted at the BglII and SpeI sites of pDM304_ *act15p* :Electra2-(GGS) _2_ -MCS. For transformation, *D. discoideum* Ax4 cells were electroporated with 1 µg plasmid DNA following the standard protocol (Nellen et al., 1984). Transformants were selected and maintained in modified HL5 growth medium containing 10 µg/mL G418.

**Cell culture, development, and labeling**

Cells were grown in modified HL5 medium at 22 ºC either in petri dishes or shake flasks. The growth medium contained 10 µg/mL G418 where appropriate. To image the vegetative stage, cells were collected, washed twice, and resuspended in phosphate buffer (PB: 12 mM KH _2_ PO _4_ , 8 mM Na _2_ HPO _4_ , pH 6.5). An aliquot of the cell suspension was plated directly onto a 24×50 mm coverslip (Matsunami). The cells were allowed to attach to the glass surface before observation. To image the slugs, washed cells were suspended at a density of 2×10 ^7^ cells/mL in PB, and 5 µL of the cell suspension was deposited on an agar plate (2% agar (Bacto) in Milli-Q water). After 15–18 h, samples were excised together with the agar sheet and placed upside down onto a φ25 mm round coverslip (Matsunami) mounted on a metal chamber (Attofluor, Invitrogen) with a 50 µm height spacer ring made of polyethylene terephthalate (vinyl patch transparent Ta-3N, Kokuyo). The inner space of the ring was filled with liquid paraffin (Nacalai Tesque, Light 26132) before observation (Hashimura et al *.* , 2024).

**Image acquisition and analysis**

The cells were observed using an inverted microscope (IX83, Olympus) equipped with a multibeam confocal scanning unit (CSU-W1, Yokogawa) and CMOS cameras (ORCA-Fusion BT, Hamamatsu). A 405 nm laser was used for excitation, together with a multiband dichroic mirror (T405/488/561/640 nm) and a 447/60 bandpass filter. Images were acquired in a 16-bit format and stored as TIFF files. Images were analyzed using ImageJ and R statistical packages. For the quantitative measurement of the mean fluorescence intensity in individual cells, cell masks were generated by binarizing fluorescence images with a threshold intensity of 430, which was set slightly above the background value of 415. The mean fluorescence intensity of the cell regions was measured per mask. To quantify the fluorescence signal in slugs, the mean fluorescence intensity of a 50 µm ^2^ rectangular region at the anterior or posterior region of the slug was measured. In all statistical analyses, *P* values were determined using the Wilcoxon rank-sum test with Bonferroni adjustment.

## Reagents

**Plasmids**

**Table:** 

Plasmid ID	Plasmid name	Description	Origin	Available from
–	pDM304	Empty vector	(Veltman et al., 2009)	NBRP-nenkin
#HH343	pDM304_ *act15p* :mTagBFP2-(GGS) _2_ -MCS	extrachromosomal vector, N-terminal mTagBFP2 tagging	(Hashimura et al., 2024)	NBRP-nenkin
#HH689	pDM304_ *act15p* :MCS-(GGS) _2_ -Electra2	extrachromosomal vector, C-terminal Electra2 tagging	This work	NBRP-nenkin
#HH675	pDM304_ *act15p* :Electra2-(GGS) _2_ -MCS	extrachromosomal vector, N-terminal Electra2 tagging	This work	NBRP-nenkin
#HH710	pDM304_ *act15p* :HistoneH1-Electra2	extrachromosomal vector for expression of HistoneH1-Electra2	This work	NBRP-nenkin
#HH695	pDM304_c *oaAp* :PH _Akt_ -Electra2	extrachromosomal vector for expression of PH _Akt_ -Electra2	This work	NBRP-nenkin
#HH677	pDM304_ *act15p* :Electra2-Lifeact	extrachromosomal vector for expression of Electra2-Lifeact	This work	NBRP-nenkin

**Primers**

**Table:** 

Primer	Sequence (5′->3′)	Used for
Electra2_fwd	AATAAAAATCAGATCCAAAAAATGGTAAGTAAAGGAGAAG	#HH675
Electra2-(GGS) _2_ _rev	AACTAGTACTAGATCTACTACCTCCTGAACCACCTTTAAATAATTCATCCATACC	#HH675
(GGS) _2_ -Electra2_fwd	CAGATCTAGTACTAGTGGTGGTTCAGGAGGTAGTGTAAGTAAAGGAGAAGAATTAATC	#HH689
Electra2 _rev	TATTTATTTAACTAGATTTAAATAATTCATCCATACC	#HH689
Lifeact_fwd	GATCTATGGGTGTCGCTGACCTGATAAAGAAGTTTGAAAGCATCTCCAAGGAAGAGA	#HH677
Lifeact_rev	CTAGTCTCTTCCTTGGAGATGCTTTCAAACTTCTTTATCAGGTCAGCGACACCCATA	#HH677
